# Supplementary material for: WordCommentsAnalyzer: A windows software tool for qualitative research
Source: F1000Res. 2018 Sep 4;7:536. Originally published 2018 May 3. [Version 2] doi: 10.12688/f1000research.14819.2 (PMC6137411; doi:10.12688/f1000research.14819.2)
Supplement: Supplementary file 3 [file f1000research-7-17614-s0002.tgz › b2a79496-4ada-47c4-9682-a99ce867836d.zip!/Eight Iranian Health Journals/Archives of Rehabilitation_73.docx]

© Archives of Rehabilitation. This work is licensed under CC BY-NC 4.0 (https://creativecommons.org/licenses/by-nc/4.0/).

Volume & Issue: دوره 1 - شماره 1

Title: اصول و قواعد توانبخشی در بیماریهای اعصاب و نورولوژیکی در سالمندی

http://rehabilitationj.uswr.ac.ir/article-1-503-fa.html

واژه توانبخشی در راستای فعالیت های پزشکان و دیگر اعضای تیم توانبخشی فقط چندی صباحی است که در مورد سالمندان بکار می رود، در سالهای 1990 در امریکا مراکز توانبخشی و یا حتی در نوعی مراکزSub-awite  توانستند با درنظرگرفتن توانایی های سالمندان به جای ناتوانی های آنها، از یک پدیده به ظاهر منفی، فرایندی کاملاً مثبت بوجود آورند. در این مقاله راه‌کارهای توانبخشی در سالمندی را مرور خواهیم کرد. علاوه‌برآن، بر انواع متدهای توانبخشی در رابطه با چند نوع بیماری اعصاب و نورولوژیک سالمندان که درجه پدیداری بالایی دارند، مروری هرچند مختصر خواهیم داشت.

Title: حافظه و تاثیر آن بر عملکرد سالمندان

http://rehabilitationj.uswr.ac.ir/article-1-504-fa.html

قابلیتهای حافظه یعنی مکانیزمها و راهبردهای حافظه با افزایش سن رو به کاهش می رود، اما تحقیقات نشان می دهد که محتوای حافظه یعنی دانش انباشته شده معمولا افزایش می یابد. سیستم حافظه شامل حافظه حسی است که اطلاعات محیطی در آن ثبت و ضبط می‌شود، حافظه کوتاه مدت برای نگاهداری اطلاعات در سطح آگاهی و حافظه دراز مدت که مربوط به ذخیره دانش و تجارب گذشته است. عوامل فیزیولوژیکی و روان‌شناختی مانند سلامت جسمانی و روانی، تغذیه، استفاده از مواد مخدر و الکل و نیز انگیزش در طول فرایند زندگی ممکن است بر کلیه عملکردهای افراد سالمند از جمله حافظه آنها اثر بگذارد. متاسفانه بسیاری از پژوهشگران در مطالعات خود توجهی به اثرات این متغیرها بر آزمودنیها نشان نداده اند.

Title: بررسی اپیدمیولوژیک افسردگی در دانشجویان پزشکی

http://rehabilitationj.uswr.ac.ir/article-1-505-fa.html

هدف: هدف از انجام این پژوهش یافتن درصد فراوانی افسردگی، شدت آن و نیز ارتباط آن با استرس‌های حاد ومزمن، سابقه بیماری‌های عصبی-روانی و برخی از شاخص‌های جمعیت شناختی در میان دانشجویان پزشکی دانشگاه بندرعباس در سال 74-1373 بود. روش: تعداد 300 پرسشنامه بر اساس نمونه‌گیری طبقه‌ای-تصادفی و با رعایت تناسب بین دوجنس در جمعیت دانشجویان در میان آنان توزیع شد و تحت بررسی قرار گرفت. یافته‌ها: در این تحقیق درصد فراوانی افسردگی و شدت آن در 271 نفر از دانشجویان دانشکده پزشکی بندرعباس با استفاده از آزمون یک مورد بررسی قرار گرفت. %1/44 از دانشجویان، افسردگی نشان دادند. شدت افسردگی در %3/26 خفیف، در %4/10 متوسط و در %4/7 شدید ارزیابی شد. فراوانی افسردگی در دانشجویان سال سوم و پنجم بیشتر بود. میزان افسردگی با متأهل بودن، تجربه استرسهای حاد، ابتلا به بیماریهای مزمن و ابتلای قبلی به افسردگی و سایر اختلالات روانی، همبستگی آماری معنی‌دار مثبتی داشت عدم علاقه به رشته پزشکی و نارضایتی از شرایط فعلی نیز همبستگی مشخصی با افسردگی نشان دادند اما سن، جنس، بومی بودن، استرسهای مزمن و سابقه خانوادگی مثبت افسردگی، رابطه معنی داری با افسردگی نداشت. نتیجه‌گیری: با توجه به درصد بالای افسردگی در دانشجوین پزشکی، ضرورت توجه خاص به این گروه از لحاظ پیشگیری، شناسایی به موقع، و درمان مشخص می‌شود. کاهش استرس‌های تحصیلی از طریق زمان‌بندی مناسب دروس و امتحانات، بازنگری به روش تدریس و مطالب آموزشی و ایجاد مراکز فعال مشاوره دانشجویی می‌تواند در این راستا مثمر ثمر واقع شود. تغییر درسیستم پذیرش دانشجو، ایجاد تسهیلات رفاهی بیشتر برای دانشجویانی که ازبیماری‌های مزمن رنج می برند و نیز بالابردن سطح آگاهی علاقه‌مندان به تحصیل در رشته پزشکی در مورد آینده شغلی خود از دیگر مواردی است که می تواند تاثیر به سزایی در این زمینه داشته باشد.

Title: اختلالات همراه با سوءمصرف مواد در نوجوانان - سرند برای اختلالات بیش فعالی و نقص توجه - سلوک - افسردگی

http://rehabilitationj.uswr.ac.ir/article-1-507-fa.html

هدف: سرند اختلالات همراه (Comorbid) با سوءمصرف مواد در نوجوانان. سوءمصرف‌کننده از میان اختلالات مختلف، سه اختلال بیش فعالی و نقص توجه (ADHD)-سلوک (Conduct) و افسردگی (Depression) در نمونه ای خاص از نوجوانان ایرانی بررسی شد. روش بررسی: تعداد 33 نفر سوءمصرف‌کننده، 35 نفر بزهکار سوء‌مصرف‌کننده، 34 نفر بزهکار غیر سوءمصرف‌کننده دختر و پسر از میان نوجوانان کانون اصلاح و تربیت تهران انتخاب با تعداد 33 نفر نمونه عادی (دختر و پسر) از مدارس جنوب شهر تهران، با استفاده از پرسشنامه خودسنجی آکنبک (YSR) (آکنبک، 1991)، پرسشنامه دموگرافیک و تاریخچه مصرف دارو (طراحی‌شده توسط پژوهشگران) بررسی شدند. یافته‌ها: در اختلال بیش فعالی و نقص توجه، بین دو گروه سوءمصرف‌کننده با دو گروه غیر سوءمصرف‌کننده، تفاوت معنا دار وجود داشت، اما بین پسران و دختران، تفاوت معنادار نبود. در اختلال سلوک، تفاوت معنادار بین دو جنس وجود داشت. در پسران، گروه های سوءمصرف‌کننده با عادی تفاوت معنادار داشتند. در دختران، بزهکاران سوءمصرف‌کننده با سه گروه دیگر تفاوت معنادار داشتند. در اختلال افسردگی، تفاوت بین دو جنس معنادار بود: در پسران، سه گروه انتخابی از کانون اصلاح و تربیت، با گروه عادی تفاوت داشتند و در دختران، سوءمصرف‌کنندگان با بزهکاران و گروه عادی تفاوت معنادار داشتند. نتیجه‌گیری: به نظر می آید سه اختلال، دارای الگوی جنسیتی متفاوتی در بین گروه ها باشند. ADHD در هر دو جنس یکسان بود. در اختلال سلوک و افسردگی، بین دو جنس تفاوت معنادار وجود داشت. در هر دو جنس، اختلال ADHD با سوءمصرف مواد همراه (Comorbid) نبود. اختلال سلوک با سوءمصرف مواد در هر دو جنس همراه نبود و اختلال افسردگی تنها در دختران با سوءمصرف مواد همراه بود. به نظر می آید با توجه به تست خودسنجی نوجوانان آکنبک (YSR) پروفایل روانی خاصی برای سوءمصرف کنندگان وجود دارد که آنها را از غیر سوءمصرف‌کننده جدا می کند.

Title: بررسی رابطه ویژگیهای خانواده با سلامت روانی دانش آموزان دختر سال سوم تجربی تهران

http://rehabilitationj.uswr.ac.ir/article-1-508-fa.html

هدف: تشکیل شخصیت پدیده پیچیده ای است. عوال متعدد داخلی و خارجی مانند زمینه های ارثی، شرایط هنگام تولد، رویدادهای بعد از تولد، تفاوتهای فردی، رفتارهای تقویت کننده مثبت و منفی و دیگر عوامل اجتماعی و فرهنگی، هر یک به تنهایی یا به صورت متعامل در شکل گیری شخصیت فرد مؤثرند. بر اساس نظریه مکتب تحلیل روانی، تجارب دوران کودکی، مخصوصاً تعامل با والدین دارای اثر غیر قابل تردیدی بر تصویر شخصیت فرد در بزرگسالی است. هدف آن بررسی فرضیه های عدم ارضای نوجوانان دانش آموز در محیط خانواده های نابسامان و ارتباط آن با اضطراب، افسردگی، پرخاشگری، وسواس و... می باشد. روش بررسی: تحقیق حاضر از نوع مورد شاهدی است و آزمودنی ها 80 نوجوان دختر دانش آموز سال سوم تجربی دبیرستانهای تهران هستند که نیمی از آنان بعنوان گروه مورد و نیمی دیگر بعنوان گروه شاهد انتخاب شده اند. گزینش آزمودنی ها با روش غربالگری و به کمک آزمون اطلاعاتی صورت گرفته است و از آزمون 90-SCL برای بررسی فرضیه ها استفاده شده است. یافته‌ها: نتایج حاصل نشان دهنده تفاوت معنی داری بین گروه های مورد و شاهد می باشد. نتیجه‌گیری: به عنوان نتیجه گیری می توان گفت،‌ فضای گروه کنترل شادتر از گروه آزمایش بود. اعضای گروه آزمایش معمولاً با یکدیگر از مسائل شان صحبت نمی کردند.

Title: منابع استرس شغلی گروهی از کارکنان سازمان بهزیستی و رابطه این استرسورها با میزان رضایت شغلی آنان

http://rehabilitationj.uswr.ac.ir/article-1-509-fa.html

هدف: این مطالعه به منظور بررسی منابع استرس (استرسورهای) شغلی گروهی کارکنان سازمان بهزیستی و همچنین رابطه این استرسورها با میزان و چگونگی رضایت شغلی آنان انجام گرفت. از منابع استرس شغلی که به طور کلی شامل دو دسته منابع یا متغیرهای فردی و منابع یا متغیرهای محیطی می باشند، احساس کنترل (منابع فردی)، تعارض نقش و ابهام نقش (منابع محیطی) مورد مطالعه قرار گرفتند. سپس رابطه هریک از این عوامل استرس آور در شغل با خشنودی کارکنان از شغلشان (در ابعاد خشنودی از ماهیت کار، سرپرست، همکاران، ترفیعات، حقوق) بررسی گردید. روش بررسی: جامعه آماری پژوهش شامل روان شناسان شاغل در سازمان بهزیستی بود که تعداد 77 نفر از آنها با روش نمونه گیری تصادفی سیستماتیک (30 درصد) برای مطالعه انتخاب شدند. داده های پژوهش با آزمون شاخص توصیف شغلی J.D.I  (اسمیت و همکاران، 1969) و پرسشنامه دانش و مهارت شغلی و همچنین چک لیست سنجش نگرش به نقش شغلی جمع آوری شدند. یافته ها: نتایج نشان دادند ، تعارض نقش و ابهام نقش که خود از عدم کفایت دانش و مهارت شغلی کارکنان است ، از عمده ترین منابع استرس شغلی کارکنان است و وجود احساس کنترل، به طور مشخص نقش تعدیل کننده ای را در این ارتباط ایفا می نماید. نتیجه گیری: تضاد نقشی و ابهام نقش،‌ تحت تاثیر مهارت های شغلی و دانش کارکنان است،‌ از طرف دیگری نظام کنترل کارمندان بر استرس شغلی ایشان تاثیر می گذارد.

Title: گزارش دو مورد سندرم X شکننده همراه با اختلال بیش فعالی-کمبود توجه و تمرکز

http://rehabilitationj.uswr.ac.ir/article-1-510-fa.html

نام سندرم X شکننده برگرفته از یک نقطه شکننده (Constriction) قابل تشخیص بر کروموزوم (در محل 27/3 xq) در محیط کشت کروموزومی بدون اسید فولیک می باشد. قسمت بدون ثبات شامل تکرار تری‌نوکلئوتیدی است که در نسلهای بعدی تشدید می شود (آمپلیفیکاسیون DAN) و موجب بروز فتوتیپ شدیدتری در فرد می شود. %20 مردان دچار سندرم X شکننده طبیعی هستند. دختران این افراد دارای کروموزوم غیر عادی خواهند بود (ناقلین) و نوه های دختر و پسرشان با علامت خواهند شد. در سندرم تی‌پیک پسران مبتلا دچار عقب ماندگی ذهنی، ماکروسفالی، گوشهای بزرگ و برجسته، صورت دراز و بزرگی بیضه ها می باشند. از نظر وجود اختلالات رفتاری همراه، وجود علایم مشابه اختلالات نافذ رشد مثل اوتیسم، اختلال توجه و تمرکز/بیش فعالی (ADHD) ذکر گردیده است. اشکال توانایی‌های شناختی به صورت مشکلات یادگیری تا اشکالات شدید مطرح شده است. بیماران ما 2 مورد (یک پسر 6 ساله و یک پسر 7 ساله ) بودند که به دلیل پر تحرکی مراجعه کردند. در معاینه فیزیکی وجود علایم اختلال توجه و تمرکز/بیش فعالی (ADHD) همچنین علایم سندرم X شکننده تأیید شد. در آزمایش کشت کرموزومی نیز وجود شکنندگی در محل 27/3 xq مشخص گردید.
